# Undiagnosed Long COVID-19 in China Among Non-vaccinated Individuals: Identifying Persistent Symptoms and Impacts on Patients' Health-Related Quality of Life

**DOI:** 10.1007/s44197-022-00079-9

**Published:** 2022-11-24

**Authors:** Lin Zhang, Jie Lei, Jintao Zhang, Longlong Yin, Yanjiao Chen, Yan Xi, Joaquim Paulo Moreira

**Affiliations:** 1 Department of Epidemiology, School of Public Health, Cheeloo College of Medicine, Shandong University, Jinan, China; 2 Shandong Center for Disease Control and Prevention, Jinan, China; 3 Department of Respiratory Diseases, Shandong Provincial Qianfoshan Hospital, Cheeloo College of Medicine, Shandong University, Jinan, China; 4 Center for Social Work and Social Governance, Department of Sociology, Henan Normal University, Xinxiang, China; 5 International Healthcare Management Research and Development Center (IHM-RDC), Shandong Provincial Qianfoshan Hospital, Jinan, China; 6 Atlantica Instituto Universitario, Gestao em Saude, Oeiras, Portugal

**Keywords:** Long COVID-19, SARS-CoV-2, Undiagnosed condition, China Health-related quality of life, HRQoL, Non-vaccinated

## Abstract

Is Long COVID-19 under-diagnosed? The definition of this new condition has received many contributions, and it is still under development as a great variety of symptoms have been associated to it. This study explores the possibility that there are non-diagnosed cases among individuals who have been infected by SARS-CoV-2 and have not been vaccinated. The long-term symptoms identified among a sample 255 individuals have been associated to Long COVID-19 by recent literature. The study relates these symptoms to risk factors and health-related quality of life (HRQoL) negative impacts. The individuals were screened 1 year after discharge to explore its potential relation to Long COVID-19. Patients diagnosed with COVID-19 and discharged from designated hospitals in a Chinese province between January and April 2020 were included in this study. They received computed tomography (CT) scans one month after discharge. One year after discharge, patients were invited to physical examination and interviewed with questionnaire on health-related quality of life (HRQoL) and post-COVID-19 symptoms. Tobit regression and Logistic regression were applied to evaluate the risk factors for health utility value and pain/discomfort and anxiety/depression. One year after discharge, 39.61% patients complained of several of the symptoms associated to Long COVID-19. More than half had abnormal chest CT. Previous studies focused on the post-COVID-19 symptoms and chest CT findings of patients, but few studies have assessed the COVID-19-associated risk factors for health-related quality of life.

## Introduction

The outbreak of COVID-19 at the end of 2019 had a significant impact on global health and economic development. By the end of May 2022, the total number of confirmed cases in the world has reached over 524 million and the number of deaths associated with SARS-CoV-2 is more than 6 million [1]. For patients who have been infected by the Severe Acute Respiratory Syndrome Coronavirus 2 (SARS-CoV-2), most cured patients can basically return to their life-style before infection.

However, post-acute sequelae of COVID-19 (PASC), also known as Long COVID-19 has become a concern in the field of public health. PASC refers, therefore, to the persistent COVID-19 symptoms after SARS-CoV-2 infection, and these symptoms indicate a long-term impact of the virus [2]. Recent research, published in 2022, clarifies the diversity of symptoms and the difference between published scientific definitions of Long COVID-19 [3].

For this study, the considered definitions of Long COVID-19 include that of a condition where individuals have any symptoms persisting for at least 1 year or persisting symptom during the follow-up time [4]. This observation is accepted as post-COVID-19 syndrome and referred to the related symptoms and sequelae due to long-term impact of COVID-19 [5].

PASC is considered as chronic sequelae persistent due to unknown causes after SARS-CoV-2 infection [6]. Long COVID-19 also includes a series of physical and mental/psychological long-term symptoms [2, 7, 8]. In October 2021, the World Health Organization named it post-COVID-19 condition and defined it as a condition in which symptoms that occurred within three months after the onset of COVID-19, lasted more than two months and could not be explained by other diagnosis. These symptoms might occur after the initial cure of COVID-19 or might be persistent from the initial disease and might fluctuate or recur as time went on [9]. At present, the clinical symptoms of SARS-CoV-2 infection in the acute phase have been widely studied and a consensus has been reached of the broad nature of this condition. However, the long-term impacts of SARS-CoV-2 infection are still being studied and systematically described [3, 55–60]. It has been suggested that SARS-CoV-2 infection will cause sequelae of immune system, blood system, pulmonary system, cardiovascular system, gastrointestinal system, musculoskeletal system, nervous system [10, 11]. Immune abnormality/imbalance may be related to post-COVID-19 syndrome [11, 12]. In addition, autoimmunity, metabolism changes, transient receptor potentiation channel dysfunction and autonomic dysfunction have also been found in patients with post-COVID-19 symptoms [11].

Evidence from previous studies suggests a diversity of impacts on the health status of patients and the exercise ability of some SARS patients has been identified as significantly lower than that of the general population, as registered 1 year after cure [3, 13, 14, 55–60]. During the COVID-19 pandemic, the health-related quality of life of the general population was affected in different ways and extents [15, 16]. Evidence also suggests that patients' health-related quality of life is significantly affected in the short term after discharge [17]. Fatigue, headache, joint pain and loss of smell were typical persistent symptoms [18, 19]. Severe patients had significant chest CT changes and pulmonary function impairment several months after discharge [20, 21]. Infection had a certain long-term impact on patients after recovery [22, 23]. In essence, a diversity of evidence demonstrated that COVID-19 has affected physical and psychological dimensions of individual health and put pressure on health services [24–26]. In addition, psychological factors have been associated to causing symptoms and burden of post-COVID-19 [27]. In this manner, long-term impacts of SARS-CoV-2 infection deserve our attention. In addition to the perceived physical symptoms and/or changes in mental health, empirical research on patients' quality of life can, to a certain extent, reflect the long-term impact of COVID-19.

Previous studies focused on the post-COVID-19 symptoms and chest CT findings of patients. Yet, few studies have assessed COVID-19-associated risk factors and impacts on health-related quality of life [3, 20, 28–31]. Evaluation of post-COVID-19 conditions and health-related quality of life among patients with COVID-19 can provide an evidence-based basis for public health decisions and healthcare management interventions.

In short, the main purpose of this study was to generate evidence on impacts on health-related quality of life faced by patients previously infected with SARS-CoV-2 in the context of the international scientific debate on post-COVID-19 persistent symptoms and Long COVID-19. One additional important note is that all patients included in the study were non-vaccinated.

## Methods

### Study Design and Participants

A total of 255 patients who were diagnosed with COVID-19 and discharged from designated hospitals in China Shandong province between January and April 2020 participated in this study. Infected patients (symptomatic and asymptomatic) were admitted to designated hospitals. At the time of follow-up, the patient had not been vaccinated against COVID-19. According to the clinical severity during acute onset, symptomatic patients were divided into mild, moderate, severe and critical [32]. All patients met discharge criteria on the basis of clinical guidelines for COVID-19 pneumonia diagnosis and treatment issued by the National Health Commission of the People's Republic of China (including 3 days without fever, improvement in respiratory symptoms, obvious acute pulmonary lesions recovery by CT imaging and negative results for nucleic acid tests with an interval at least 24 h) [32]. The following patients were not included in this study: (1) patients who could not participate due to hospitalization or locomotion difficulties (2) patients who refused to participate, (3) patients who could not be contacted, (4) patients who left Shandong province for personal reasons such as job changes. This study was approved by the ethics committee of Shandong Center for Disease Control and Prevention and all research processes met ethical standards. Flowchart is shown below.
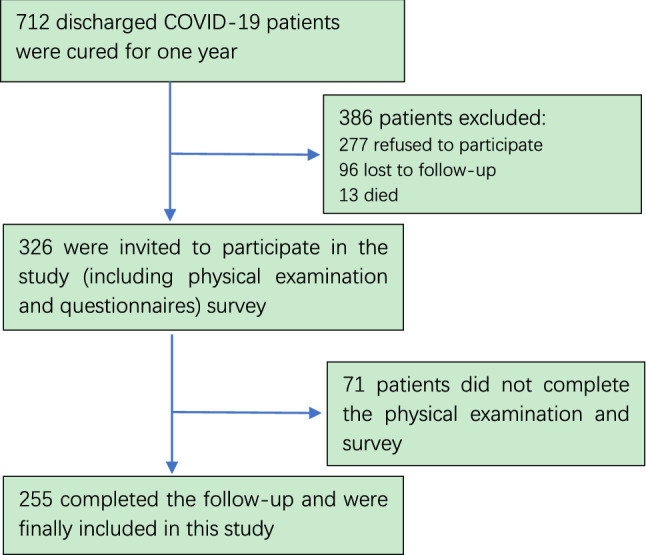


### Data Collection

#### Data Collection at Acute Phase

We collected patients' baseline data including age, sex, clinical severity during acute onset, date of onset, admission hospitals, date of discharge from electronic medical system. None of the patients included had been vaccinated.

#### Follow-up Assessment

One month after discharge, patients were invited to have a computed tomography (CT) scans to observe the early recovery. The routine follow-up 1 year after discharge was conducted between March 18, 2021 and April 25, 2021. Detailed follow-up data were obtained from physical examination based on computed tomography (CT) scans and series of questionnaires to reveal post-COVID-19 symptoms based on the health-related quality of life (HRQoL) scale. Face-to-face interviews or telephone interviews were conducted by uniformly trained investigators to complete the questionnaires to collect data on the post-COVID-19 symptoms (including fatigue, dyspnea, joint/muscle pain, low fever, difficulty attention, memory problems, sleep disorders, rash or hair loss, palpitation, chest pain, cough, smell/taste disorders) and health-related quality of life factors. Post-COVID-19 symptoms were defined as persistent symptoms that appeared after SARS-CoV-2 infection or persistent symptoms worse than before the infection. In addition, the working status and social aspects influenced by SARS-CoV-2 infection were also taken into account in the questionnaire. We controlled the missing rate within 10% and applied multiple imputation for the few missing data.

### Measurement

#### Health-Related Quality of Life

The questionnaire was designed to measure the health status of cured patients, including sociodemographic information, post-COVID-19 symptoms after discharge and the five-dimensional health-related quality of life (HRQoL) items included in the instrument (EQ-5D-5L). In the first half of 2021, most of the cured patients and asymptomatic infected people in the early stage of the COVID-19 in Shandong Province have been discharged from the hospital for nearly a year. Our approach to data gathering aimed to describe the physical and mental health of cured patients, scientifically evaluate the curative and rehabilitation developments, as well as fully understand the self-evaluation of patients on their own health status and the influencing factors. This process provided an evidence-based evidence basis for further improving the treatment mechanism and patient care associated with infectious diseases and public health emergencies.

Additionally, we should clarify that PRO (patient-reported outcomes) is an approach directly derived from patients' own perceptions on health status and functional status. I has been widely used in clinical research. The outcomes reported by the patients mainly include symptoms, health-related quality of life (HRQoL) perceptions as well as psychological distress and self-efficacy [33]. HRQoL has been used to measure the health status of individuals under the influence of diseases and injuries, clinical intervention, aging and social environment changes [15, 16, 34, 35]. Currently, COVID-19 patient's self-perceived health after discharge requires ongoing evidence. As an additional result to assess the final consequences of COVID-19 on physical health and functional conditions, patient-centered studies on Long COVID-19 are helpful for personalized rehabilitation care following COVID-19 infection [36].

Health-related quality of life (HRQoL) data in this study were collected by applying the EQ-5D-5L. EuroQol (EQ-5D-5L) is a widely used scale to evaluate health-related quality of life (HRQoL) and includes five dimensions (mobility, self-care, usual activities, pain/discomfort, anxiety/depression). Each dimension is evaluated through five levels from no problems to extreme problems (level 1 = no problems, level 2 = slight problems, level 3 = moderate problems, level 4 = severe problems, level 5 = extreme problems). Based on the health preference of the population under study, EQ-5D-5L health utility value (range − 0.391 – 1.000) was calculated through the value set established by the time trade-off (TTO) method [37]. The self-health evaluation was intuitively assessed by EuroQol Visual Analog Scale (EQ-VAS). Respondents were asked to indicate a number on the scale to represent their perceived health level (i.e.: scale 0–100, subjective self-health evaluation). The higher the health utility value and self-health evaluation, the better the patients' health condition [38] was perceived.

#### Independent Variable

The Tobit regression was applied. The independent variables of the study included age range, sex, cigarette smoking (including non-smoker, former smoker, current smoker), clinical severity (including asymptomatic, mild, moderate, severe/critical), number of post-COVID-19 symptoms and chest CT abnormal findings including fibrous stripe, pleural thickening/adhesion, single nodule, multiple nodules and ground glass opacity (GGO). Considering the sample size, the independent variable is simplified as age, sex, cigarette smoking (including non-smoker, former smoker, current smoker), clinical severity (including asymptomatic, mild, moderate, severe/critical), at least one CT abnormal finding, at least one post-COVID-19 symptom in logistic regression.

### Statistical Analysis

Patients with severe and critical complaints were combined into one group for analysis. Continuous variables were described as mean standard deviation. Categorical variables and ranked ordinal variables were described as frequency (percentage). Both Shapiro–Wilk test and Levene’s test were applied to test the normality and homogeneity of variance for continuous variables. Comparison between groups was performed by one-way ANOVA or Kruskal–Wallis test. Kruskal–Wallis test was also used for comparison of ranked ordinal variables; *χ*^2^ test or Fisher’s exact test which were used for comparison of categorical variables when appropriate. McNemar's test was used for comparison of longitudinal chest CT results.

As commonly practiced, Tobit regression was applied to assess risk factors for health utility value [34, 39]. Logistic regression was performed to explore the risk factors for pain/discomfort and anxiety/depression. All statistical analyses were conducted using R software (version 4.0.3). The statistical significance level was set at *p* < 0.05 with two-tailed.

## Results

Table 1 shows the demographic characteristics, post-COVID-19 symptoms and chest CT results of cured patients 1 year after discharge. Of all patients, 125 (49.02%) were female and 130 (50.98%) were male. The average age was 44 ± 16 years. Compared with other groups, patients with severe/critical symptoms were much older (*p* < 0.05). It was shown that 39.61% of the patients included in the study had post-COVID-19 symptoms. Fatigue was the most common persistent symptom, 17.65% of patients suffered from this symptom. Furthermore, a considerable number of the patients suffered from sleep disorders, joint/muscle pain and dyspnea, namely 15.69%, 11.76% and 9.02% respectively. Of the 255 patients who participated in the follow-up, 10 refused to participate in the chest CT examination. CT results showed that more than half (55.92%) of the patients had abnormal chest CT findings. There were 45 cases of fibrous stripe (18.37%) and the proportions of fibrous stripe in severe/critical patients was the highest (*p* < 0.05). Other findings, such as single nodule (13.88%) and multiple nodules (8.98%), also showed a relevant proportion. In addition, 13 cases (5.31%) were diagnosed as pleural thickening/adhesion and GGO, respectively.

**Table 1:** Demographic characteristics, post-COVID-19 symptoms and chest CT results of patients

Characteristic	Asymptomatic *n* = 46	Mild *n* = 35	Moderate *n* = 166	Severe/Critical *n* = 8	Total *n* = 255	*p* value
Sex, * n* (%)						
Women	21 (45.65)	16 (45.71)	84 (50.60)	4 (50.00)	125 (49.02)	
Men	25 (54.35)	19 (54.29)	82 (49.40)	4 (50.00)	130 (50.98)	0.912
Age (years), mean ± sd	40.61 (19.88)	39.83 (19.17)	44.81 (13.81)	58.12 (12.8)	43.78 (16.08)	0.011
Age range (years), * n* (%)						
0–17	8 (17.39)	4 (11.43)	5 (3.01)	0 (0.00)	17 (6.67)	
18–44	15 (32.61)	15 (42.86)	79 (47.59)	1 (12.50)	110 (43.14)	
45–59	16 (34.78)	10 (28.57)	58 (34.94)	4 (50.00)	88 (34.51)	0.114
≥ 60	7 (15.22)	6 (17.14)	24 (14.46)	3 (37.50)	40 (15.69)	
Cigarette smoking, * n* (%)						
Non-smoker	38 (82.61)	34 (97.14)	141 (84.94)	7 (87.50)	220 (86.27)	
Former smoker	1 (2.17)	0 (0.00)	8 (4.82)	1 (12.50)	10 (3.92)	
Current smoker	7 (15.22)	1 (2.86)	17 (10.24)	0 (0.00)	25 (9.80)	0.232
Employment status, * n* (%)						
Full-time job	20 (43.48)	19 (54.29)	92 (55.42)	3 (37.50)	134 (52.55)	
Retired	6 (13.04)	7 (20.00)	18 (10.84)	3 (37.50)	34 (13.33)	
Full-time student	9 (19.57)	3 (8.57)	6 (3.61)	0 (0.00)	18 (7.06)	
Jobless	11 (23.91)	6 (17.14)	50 (30.12)	2 (25.00)	69 (27.06)	0.691
Post-COVID-19 symptoms, * n* (%)						
At least one symptom	12 (26.09)	14 (40.00)	71 (42.77)	4 (50.00)	101 (39.61)	0.206
Fatigue	6 (13.04)	5 (14.29)	32 (19.28)	2 (25.00)	45 (17.65)	0.672
Dyspnea	1 (2.17)	7 (20.00)	14 (8.43)	1 (12.50)	23 (9.02)	0.039
Joint/muscle pain	4 (8.70)	4 (11.43)	21 (12.65)	1 (12.50)	30 (11.76)	0.888
Low fever	0 (0.00)	0 (0.00)	2 (1.20)	0 (0.00)	2 (0.78)	> 0.999
Difficulty attention	2 (4.35)	1 (2.86)	6 (3.61)	0 (0.00)	9 (3.53)	0.910
Memory problems	3 (6.52)	1 (2.86)	15 (9.04)	1 (12.50)	20 (7.84)	0.531
Sleep disorders	5 (10.87)	7 (20.00)	26 (15.66)	2 (25.00)	40 (15.69)	0.610
Rash or hair loss	4 (8.70)	1 (2.86)	12 (7.23)	0 (0.00)	17 (6.67)	0.769
Palpitation	0 (0.00)	4 (11.43)	8 (4.82)	1 (12.50)	13 (5.10)	0.051
Chest pain	1 (2.17)	3 (8.57)	8 (4.82)	1 (12.50)	13 (5.10)	0.337
Cough	2 (4.35)	2 (5.71)	12 (7.23)	0 (0.00)	16 (6.27)	0.955
Smell/taste disorders	4 (8.70)	1 (2.86)	9 (5.42)	0 (0.00)	14 (5.49)	0.741
Number of post-COVID-19 symptoms, * n* (%)						
0	34 (73.91)	21 (60.00)	95 (57.23)	4 (50.00)	154 (60.39)	
1–2	7 (15.22)	8 (22.86)	44 (26.51)	2 (25.00)	61 (23.92)	
3–4	3 (6.52)	5 (14.29)	18 (10.84)	2 (25.00)	28 (10.98)	
≥ 5	2 (4.35)	1 (2.86)	9 (5.42)	0 (0.00)	12 (4.71)	0.253
Chest CT abnormal findings, * n* (%)						
At least one CT abnormal finding	23/46 (50.00)	18/31 (58.06)	90/161 (55.90)	6/7 (85.71)	137/245 (55.92)	0.357
Fibrous stripe	5/46 (10.87)	8/31 (25.81)	26/161 (16.15)	6/7 (85.71)	45/245 (18.37)	< 0.001
Pleural thickening/adhesion	0/46 (0.00)	2/31 (6.45)	11/161 (6.83)	0/7 (0.00)	13/245 (5.31)	0.251
Single nodule	7/46 (15.22)	4/31 (12.90)	23/161 (14.29)	0/7 (0.00)	34/245 (13.88)	0.911
Multiple nodules	3/46 (6.52)	8/31 (25.81)	11/161 (6.83)	0/7 (0.00)	22/245 (8.98)	0.018
GGO	2/46 (4.35)	2/31 (6.45)	9/161 (5.59)	0/7 (0.00)	13/245 (5.31)	0.942
Consolidation	0/46 (0.00)	0/31 (0.00)	1/161 (0.62)	0/7 (0.00)	1/245 (0.41)	> 0.999
Others	11/46 (23.91)	4/31 (12.90)	28/161 (17.39)	0/7 (0.00)	43/245 (17.55)	0.356

*GGO* ground glass opacity

Table 2 shows the health-related quality of life identified. The average health utility value was 0.94 ± 0.12, and the average self-health evaluation score was 81.89 ± 14.76. Most patients recovered well and report high quality of life. As also shown in the appendix table, compared with patients without post-COVID-19 symptoms and CT abnormalities, the health utility value of patients with CT abnormalities, or post-COVID-19 symptoms, decreased significantly, namely 0.93 ± 0.15 and 0.90 ± 0.16 respectively. In addition, the patients with both post-COVID-19 symptoms and CT abnormalities show the lowest health utility value, which was only 0.88 ± 0.20. Few patients had problems in the three dimensions of mobility, self-care and usual activities. However, 52 patients (20.39%) reported different levels of pain/discomfort. 81 patients (31.76%) reported different levels of anxiety/depression. For these two dimensions, with a considerable proportion of abnormal conditions, we used logistic regression to explore the further explore associated risk factors.

**Table 2:** Health-related quality of life of COVID-19 patients

Characteristic	Asymptomatic *n* = 46	Mild *n* = 35	Moderate *n* = 166	Severe/Critical *n* = 8	Total *n* = 255	*p* value
Health utility value, mean ± sd	0.97 ± 0.06	0.94 ± 0.08	0.94 ± 0.13	0.88 ± 0.17	0.94 ± 0.12	0.429
Self-health evaluation, mean ± sd	83.87 ± 14.78	81.54 ± 14.02	81.57 ± 14.69	78.75 ± 20.31	81.89 ± 14.76	0.703
Mobility, * n* (%)						
No problems	45 (97.83)	32 (91.43)	150 (90.36)	6 (75.00)	233 (91.37)	
Slight problems	1 (2.17)	1 (2.86)	11 (6.63)	1 (12.50)	14 (5.49)	
Moderate problems	0 (0.00)	2 (5.71)	3 (1.81)	1 (12.50)	6 (2.35)	
Severe problems	0 (0.00)	0 (0.00)	2 (1.20)	0 (0.00)	2 (0.78)	
Extreme problems	0 (0.00)	0 (0.00)	0 (0.00)	0 (0.00)	0 (0.00)	0.142
Self-care, * n* (%)						
No problems	45 (97.83)	35 (100.00)	163 (98.19)	7 (87.50)	250 (98.04)	
Slight problems	1 (2.17)	0 (0.00)	1 (0.60)	1 (12.50)	3 (1.18)	
Moderate problems	0 (0.00)	0 (0.00)	0 (0.00)	0 (0.00)	0 (0.00)	
Severe problems	0 (0.00)	0 (0.00)	1 (0.60)	0 (0.00)	1 (0.39)	
Extreme problems	0 (0.00)	0 (0.00)	1 (0.60)	0 (0.00)	1 (0.39)	0.155
Usual activities, * n* (%)						
No problems	44 (95.65)	34 (97.14)	154 (92.77)	6 (75.00)	238 (93.33)	
Slight problems	2 (4.35)	1 (2.86)	9 (5.42)	1 (12.50)	13 (5.10)	
Moderate problems	0 (0.00)	0 (0.00)	1 (0.60)	1 (12.50)	2 (0.78)	
Severe problems	0 (0.00)	0 (0.00)	1 (0.60)	0 (0.00)	1 (0.39)	
Extreme problems	0 (0.00)	0 (0.00)	1 (0.60)	0 (0.00)	1 (0.39)	0.122
Pain/discomfort, * n* (%)						
No problems	39 (84.78)	28 (80.00)	131 (78.92)	5 (62.50)	203 (79.61)	
Slight problems	6 (13.04)	6 (17.14)	29 (17.47)	2 (25.00)	43 (16.86)	
Moderate problems	1 (2.17)	1 (2.86)	6 (3.61)	1 (12.50)	9 (3.53)	
Severe problems	0 (0.00)	0 (0.00)	0 (0.00)	0 (0.00)	0 (0.00)	
Extreme problems	0 (0.00)	0 (0.00)	0 (0.00)	0 (0.00)	0 (0.00)	0.481
Anxiety/depression, * n* (%)						
No problems	34 (73.91)	21 (60.00)	114 (68.67)	5 (62.50)	174 (68.24)	
Slight problems	10 (21.74)	10 (28.57)	34 (20.48)	1 (12.50)	55 (21.57)	
Moderate problems	1 (2.17)	3 (8.57)	12 (7.19)	2 (25.00)	18 (7.06)	
Severe problems	0 (0.00)	1 (2.86)	3 (1.81)	0 (0.00)	4 (1.57)	
Extreme problems	1 (2.17)	0 (0.00)	3 (1.81)	0 (0.00)	4 (1.57)	0.538

Tobit regression allowed to demonstrate that the health utility value was significantly correlated with age ≥ 60 (coefficient = − 0.164, 95% CI − 0.292 to − 0.036), fibrous stripe (coefficient = − 0.089, 95% CI − 0.155 to − 0.023) and reporting different numbers of post-COVID-19 symptoms (1–2 coefficient = − 0.086, 95% CI − 0.145 to − 0.027; 3–4 − 0.177, 95% CI − 0.253 to − 0.100; ≥ 5 − 0.296, 95% CI − 0.402 to − 0.191) (Table 3). However, clinical severity was not a risk factor for the health utility value. Logistic regression showed that age (OR: 1.03, 95% CI 1.01 to 1.06), sex (female) (OR: 2.96, 95% CI 1.37 to 6.83) and having at least one post-COVID-19 symptom (OR: 4.77, 95% CI 2.39 to 9.91) were risk factors for pain/discomfort. Furthermore, it is identified that having at least one post-COVID-19 symptom (OR: 3.44, 95%CI: 1.95 to 6.15) was a risk factor for anxiety/depression (Table 4).

**Table 3:** Results of Tobit regression for health utility value

Variables	Coefficient	95% CI	*p* value
Age range (years)			
0–17	Ref.		
18–44	− 0.017	(− 0.139, 0.105)	0.783
45–59	− 0.079	(− 0.200, 0.042)	0.200
≥ 60	− 0.164	(− 0.292, − 0.036)	0.012
Sex			
Men	Ref.		
Women	− 0.029	(− 0.082, 0.025)	0.299
Cigarette smoking			
Non-smoker	Ref.		
Former smoker	− 0.070	(− 0.190, 0.049)	0.248
Current smoker	− 0.006	(− 0.097, 0.086)	0.899
Clinical severity			
Asymptomatic	Ref.		
Mild	− 0.040	(− 0.134, 0.054)	0.407
Moderate	− 0.019	(− 0.088, 0.051)	0.598
Severe/Critical	0.001	(− 0.153, 0.154)	0.993
CT abnormal findings			
Fibrous stripe	0.015	(− 0.073, 0.103)	0.510
Pleural thickening/adhesion	− 0.089	(− 0.155, − 0.023)	0.009
Single nodule	0.075	(− 0.040, 0.189)	0.202
Multiple nodules	0.045	(− 0.034, 0.123)	0.266
GGO	0.038	(− 0.074, 0.150)	0.734
Number of post-COVID-19 symptoms			
0	Ref.		
1–2	− 0.086	(− 0.145, − 0.027)	0.004
3–4	− 0.177	(− 0.253, − 0.100)	< 0.001
≥ 5	− 0.296	(− 0.402, − 0.191)	< 0.001

**Table 4:** Results of logistic regression for pain/discomfort and anxiety/depression

Variables	Model 1^a^		Model 2^b^	
	OR (95%CI)	*p* value	OR (95%CI)	*p* value
Age (years)	1.03(1.01, 1.06)	0.009	1.02(1.00, 1.04)	0.073
Sex				
Men	Ref.		Ref.	
Women	2.96 (1.37, 6.83)	0.008	1.26(0.67, 2.36)	0.474
Cigarette smoking				
Non-smoker	Ref.		Ref.	
Former smoker	2.14(0.38, 10.14)	0.350	1.30(0.29, 5.35)	0.722
Current smoker	1.18(0.24, 4.59)	0.822	1.38(0.48, 3.73)	0.537
Clinical severity				
Asymptomatic	Ref.		Ref.	
Mild	1.52(0.40, 5.82)	0.536	2.22(0.79, 6.42)	0.133
Moderate	1.12(0.43, 3.25)	0.827	1.06(0.49, 2.39)	0.887
Severe/Critical	1.92(0.29, 12.24)	0.483	1.19(0.19, 6.73)	0.844
At least one CT abnormal finding	0.90(0.43, 1.89)	0.784	0.92(0.50, 1.66)	0.772
At least one post-COVID-19 symptom	4.77(2.39, 9.91)	< 0.001	3.44(1.95, 6.15)	< 0.001

*OR* odds ratio

^a^Model 1 for pain/discomfort; “have problems” in one dimension (pain/discomfort) of EQ-5D-5L

^b^Model 2 for anxiety/depression; “have problems” in one dimension (anxiety/depression) of EQ-5D-5L

Table 5 shows the impact of the disease on the working status and social contact. About a quarter of the patients (27.45%) reported that the disease affected their ability to work, possibly due to physical discomfort and persistent symptoms. 123 patients (48.24%) believed that the disease produced an effect on their working abilities. Nearly two-thirds of patients believed that the disease influenced their social participation (64.71%) or brought a sense of isolation (61.18%).

**Table 5:** Working status and social contact among COVID-19 patients

Characteristic	Asymptomatic *n* = 46	Mild *n* = 35	Moderate *n* = 166	Severe/Critical *n* = 8	Total *n* = 255	*p* value
Work ability, * n* (%)						
No impact	39 (84.78)	23 (65.71)	117 (70.48)	6 (75.00)	185 (72.55)	
A little impact	6 (13.04)	7 (20.00)	36 (21.69)	2 (25.00)	51 (20.00)	
Moderate impact	0 (0.00)	3 (8.57)	10 (6.02)	0 (0.00)	13 (5.10)	
Severe impact	1 (2.17)	2 (5.71)	3 (1.81)	0 (0.00)	6 (2.35)	0.159
Working conditions, * n* (%)						
No impact	25 (54.35)	20 (57.14)	83 (50.00)	4 (50.00)	132 (51.76)	
A little impact	10 (21.74)	7 (20.00)	34 (20.48)	3 (37.50)	54 (21.18)	
Moderate impact	3 (6.52)	5 (14.29)	28 (16.87)	1 (12.50)	37 (14.51)	
Severe impact	8 (17.39)	3 (8.57)	21 (12.65)	0 (0.00)	32 (12.55)	0.801
Social participation, * n* (%)						
No impact	22 (47.83)	10 (28.57)	55 (33.13)	3 (37.50)	90 (35.29)	
A little impact	13 (28.26)	13 (37.14)	54 (32.53)	2 (25.00)	82 (32.16)	
Moderate impact	5 (10.87)	8 (22.86)	32 (19.28)	2 (25.00)	47 (18.43)	
Severe impact	6 (13.04)	4 (11.43)	25 (15.06)	1 (12.50)	36 (14.12)	0.357
Sense of isolation, * n* (%)						
No impact	21 (45.65)	10 (28.57)	67 (40.36)	1 (12.50)	99 (38.82)	
A little impact	11 (23.91)	12 (34.29)	51 (30.72)	4 (50.00)	78 (30.59)	
Moderate impact	6 (13.04)	7 (20.00)	24 (14.46)	3 (37.50)	40 (15.69)	
Severe impact	8 (17.39)	6 (17.14)	24 (14.46)	0 (0.00)	38 (14.90)	0.462

Table 6 shows the longitudinal chest CT results of cured patients. Due to the strict epidemic prevention and control in the first half of 2020, a portion of patients refused to go to the hospital for CT examination. A total of 204 patients participated in chest CT examination twice: one month and 1 year after discharge. One month after discharge, 26.96% of the patients' lung lesions were still in the process of absorption and recovery. According to the analysis of longitudinal chest CT results, we found an increase in the proportion of fibrous stripe and single/multiple nodules. The proportion of GGO and consolidation decreased after 1 year, from 24 (11.76%) and 7 (3.43%) to 11 (5.39%) and 1 (0.49%), respectively.

**Table 6:** Longitudinal chest CT results

Characteristic	One month after discharge *n* = 204	One year after discharge *n* = 204	*p* value
At least one CT abnormal finding, * n* (%)	121 (59.31)	115 (56.37)	0.023
Inflammatory absorption, * n* (%)	55 (26.96)	/	/
Fibrous stripe, * n* (%)	14 (6.86)	42 (20.59)	< 0.001
Pleural thickening/adhesion, * n* (%)	8 (3.92)	12 (5.88)	< 0.001
Single nodule, * n* (%)	15 (7.35)	25 (12.25)	< 0.001
Multiple nodules, * n* (%)	7 (3.43)	21 (10.29)	< 0.001
GGO, * n* (%)	24 (11.76)	11 (5.39)	< 0.001
Consolidation, * n* (%)	7 (3.43)	1 (0.49)	< 0.001
Others, *n* (%)	29 (14.22)	35 (17.16)	< 0.001

## Discussion

One year after discharge, more than half of the non-vaccinated patients included in the study, had post-COVID-19 symptoms and abnormal chest CT findings. The patients' health-related quality of life was still affected by the infection of SARS-CoV-2. This study explored the risk factors for health-related quality of life (HRQoL) of patients with COVID-19 after discharge. The key risk factors explored included age, sex (female), abnormal chest CT findings and having post-COVID-19 symptoms.

Results show that public health authorities must continuously monitor post-COVID-19 impacts. Our results sustain this recommendation as also suggested by other recent international evidence. In this study, nearly two-fifths of the cured patients reported post-COVID-19 symptoms, such as fatigue, dyspnea, sleep disorders and joint/muscle pain, after cure.

The data generated in this study, one of the first undertaken in China on this topic, confirm other recent international evidence available. Namely, fatigue and dyspnea were also identified in our study as the major persistent symptoms of COVID-19 patients as identified in acute SARS patients, 1 year after infection. This was also identified in other studies [2, 4–7, 9, 14, 22, 28, 40–42]. In addition, our data indicate that patients complained of headache, joint/muscle pain and cough, which also corroborates other studies [2, 4, 6, 7]. Hence, in non-critical patients, it was found that nearly one-third suffered from these persistent symptoms [43].

Other available studies focused on the patients' mental/psychological sequelae, of which cognitive impairment was the most common problem [2, 5–7, 9]. Emotional disorders, such as anxiety/depression, plagued the patients' daily life [2, 4, 5, 7, 22, 27], which is also a set of persistent symptoms with high recurrence [44]. Our data for China are in agreement with these studies.

Other symptoms reported in other studies, including sleep disorders [4, 6, 7] and memory problems [4, 5, 7] were also complains identified in our study. In this regard, we can argue that emotional disorders and cognitive impairment might be caused by persistent inflammation. Thus, further relevant research on physiological mechanism of Long COVID-19 especially psychiatric sequelae can contribute to guide the treatment of mental disorders after COVID-19 rehabilitation, as also argued in available evidence [2].

In addition, this study suggests that SARS-CoV-2 infection had a negative impact on working status and social contact, which may aggravate patients' psychological discomfort.

Our results suggest that follow-up observations should be carried out regularly to determine the long-term effects of COVID-19 and psychological support to patients should be strengthened as a part of healthcare management programs.

This study also contributes to the notion that following SARS-CoV-2 infection, the virus invaded the lungs and causes varying degrees of lung damage. Thus, in addition to clinical diagnostic indicators, it can be argued that chest CT is also an important index for recovery evaluation. Abnormal results of chest CT are also associated as a manifestation of Long COVID-19 [5].

In line with other available evidence [42], this study also contributes to sustain the awareness that chest CT of asymptomatic patients may show a certain proportion of abnormal manifestations. Chest CT findings vary in different stages of the disease. At the acute onset, the common CT findings tend to be GGO, consolidation, crazy-paving pattern, reticular pattern, pleural thickening or adhesion [42, 45–47]. With the gradual recovery of the disease, the pulmonary lesions of the patients show fibrous stripe, single/multiple nodules, GGO and pleural thickening/adhesion. This study contributes to sustain previous studies in this regard [29, 30, 43, 48]. Single/multiple nodules may also be a permanent mark of lung injury [49], as also identified in our study. Hence, one major need evolving from this evidence, is that patients with COVID-19 deserve more attention to optimize their rehabilitation.

On what concerns the application of the EQ-5D-5L this being simple but widely used method to measure the health-related quality of life (HRQoL) among the general population according to the health utility value [37, 38], the study serves to demonstrate its applicability. In recent years, several studies have applied EQ-5D-5L to evaluate patients' health-related quality of life [15, 16, 34, 35, 50]. However, it was rarely used on patients with COVID-19.

After timely diagnosis and treatment, the average health utility value of cured patients in our study was slightly lower than that of the general population in eastern China, of which the health utility value is 0.9635 (0.9626, 0.9643) [51]. Previous studies have shown that age was a risk factor for poor prognosis [42, 52, 53]. In this study, age, sex (female) and having at least one post-COVID-19 symptom were risk factors associated with pain/discomfort. The discomfort symptoms were more likely to occur in women, which was consistent with previous studies [22, 27, 31, 54, 55].

More importantly, our study generates evidence to argue that patients' health-related quality of life did not fully return to the previous level 1 year after infection. Patients' health utility value was related to age, reporting post-COVID-19 symptoms and chest CT abnormal finding. For the near future, the transmissibility of the current or new variants and the evolution of the severity still arouses widespread concern [56]. However, whether the variants affect health-related quality of life continuously and aggravate post-COVID-19 symptoms, remains to be further studied. Considering that the current epidemic is unlikely to be completely eliminated and the adverse long COVID-19 effects, further investment in vaccination should play an important role in curbing the spread of the epidemic [57].

As with this study, several other studies on the topic of COVID-19 have found that young adult patients have been affected by Long COVID-19. This reinforces the relevance of our study, namely for the context of such large country as China.

Hence, previous studies explored the understanding of the process and perspectives of related molecular mechanism. Several sources of evidence suggest that the symptoms of long COVID-19 are similar to myalgic encephalomyelitis/chronic fatigue syndrome (ME/CFS). Additionally, several studies argue that long COVID-19 can be effectively explored through a similar pathological mechanism [11, 12, 58]. Further evidence on the molecular mechanism of long COVID-19 and its generation process needs to be generated and analyzed, including T cell depletion and interferon changes in patients [11, 59]. Longitudinal studies and follow-up evidence will be helpful to explain the pathogenesis of long COVID-19 as to provide intervention guidance for patients' rehabilitation. Results from this study contribute to this perspective.

 Available evidence generated on the large-scale vaccination changing the severity and process of the disease and an improved understanding of the clinical characteristics and persistent symptoms after the SARS-CoV-2 infection, suggest the importance of exploring, in other Future studies, relevant risk factors and prevention methods of Long COVID-19 in children, adolescents and young people [60]. However, our understanding of the long-term impact of new infectious diseases, 1 year after the acute infection period, is still insufficient. In future, we need more empirical evidence to optimize clinical diagnosis, clinical intervention and nursing care. The pathological study of COVID-19 can enrich the research progress and knowledge on chronic disabilities and disease caused by unexplained infection [5, 6]. After two years of COVID-19 pandemic, we should now face the need develop solutions to deal with growing cases of Long COVID-19 in China and in the World. From this trend, stems the current direction for research tackling the need to generate epidemiological data on long-term effects of SARS-CoV-2 [3].

This study faced two main limitations. First, the follow-up could not be traced back to all patients, because a small number of them left Shandong province due to job changes. However, the study controlled the missing rate within 10% and filled the missing data using multiple imputation. Second, this study only focused on the health status of patients 1 year after discharge. It is necessary to continue long-term monitoring and follow-up evaluation on the health impacts of SARS-CoV-2 infection.

## Conclusion

In conclusion, this study identified a worrying proportion of cured patients with post-COVID-19 symptoms and abnormal chest CT. These findings, 1 year after discharge, generate original evidence from China, concerning a phenomenon of non-diagnosed. Long COVID-19. This assumption can be ascertained from several aspects of the physical and mental conditions identified. The application of a health-related quality of life (HRQoL) instrument, generates evidence on identifying the impacts of this condition. The findings complement international available evidence on the long-term impacts of SARS-CoV-2 infection and clarify the association to negative impacts on the patients' health-related quality of life.

One key implication for health systems around the world, is that it is necessary to carry out follow-up observations and ongoing monitoring. Adopting the EuroQol (EQ-5D-5L) scale to evaluate health-related quality of life (HRQoL) on this group of patients generates relevant evidence as it includes five dimensions (mobility, self-care, usual activities, pain/discomfort, anxiety/depression) which have been widely associated to Long COVID-19.

In face of other emerging infectious diseases, clinical treatment and patient rehabilitation are two most important healthcare management and public health issues that need to be tackled by health systems and healthcare organizations around the world.

In addition to perceived physical conditions, changes in the mental health of patients cannot be ignored. This conclusion further confirms the importance of psychological support and intervention as an equally important competency for healthcare professionals and clinical decision pertaining to these patients.

Also, social aspects, such as working status and social contact, have been identified as factors affecting the emergence of Long COVID-19 cases.

In essence, besides the possible under-diagnosed phenomena identified, a matter that needs further studies, measuring patients' quality of life is conducive to fully understand the impact of Long COVID-19 and reasonably estimate the social burden of the disease to assist public health programs, optimize rehabilitation management, contribute to informed decision-making and generate improved global health.

## Data Availability

Data available on request due to restrictions (eg privacy or ethical). The data presented in this study are available on request from the corresponding author. The data are not publicly available due to Patient Privacy Protection.

## Appendix A

Health utility value of different types of patients.

**Table:**

Characteristic	Number of patients	Health utility value mean ± sd	*p* value
Without post-COVID-19 symptoms and CT abnormalities	68	0.98 ± 0.05	
Only CT abnormalities	137	0.93 ± 0.15	
Only post-COVID-19 symptoms	95	0.90 ± 0.16	
With post-COVID-19 symptoms and CT abnormalities	55	0.88 ± 0.20	< 0.001

## Appendix B

Tables 1, 2, 3, 4, 5, 6.

## Abbreviations

PASC: Post-acute sequelae of COVID-19
PRO: Patient-reported outcomes
HRQoL: Health-related quality of life
CT: Computed tomography
EQ-VAS: EuroQol Visual Analog Scale
